# A Novel Risk Model Based on Lipid Metabolism-Associated Genes Predicts Prognosis and Indicates Immune Microenvironment in Breast Cancer

**DOI:** 10.3389/fcell.2021.691676

**Published:** 2021-06-14

**Authors:** Zhimin Ye, Shengmei Zou, Zhiyuan Niu, Zhijie Xu, Yongbin Hu

**Affiliations:** ^1^Department of Pathology, School of Basic Medical Sciences, Central South University, Changsha, China; ^2^Department of Pathology, Xiangya Hospital, Central South University, Changsha, China; ^3^Department of Neurology and Research Center of Neurology in Second Affiliated Hospital, Key Laboratory of Medical Neurobiology of Zhejiang Province, Zhejiang University School of Medicine, Hangzhou, China

**Keywords:** breast cancer, lipid metabolism, tumor immune microenvironment, risk model, prognosis

## Abstract

**Background:**

Breast cancer (BRCA) is the most common tumor in women, and lipid metabolism involvement has been demonstrated in its tumorigenesis and development. However, the role of lipid metabolism-associated genes (LMAGs) in the immune microenvironment and prognosis of BRCA remains unclear.

**Methods:**

A total of 1076 patients with BRCA were extracted from The Cancer Genome Atlas database and randomly assigned to the training cohort (*n* = 760) or validation cohort (*n* = 316). Kaplan–Meier analysis was used to assess differences in survival. Consensus clustering was performed to categorize the patients with BRCA into subtypes. Using multivariate Cox regression analysis, an LMAG-based prognostic risk model was constructed from the training cohort and validated using the validation cohort. The immune microenvironment was evaluated using the ESTIMATE and tumor immune estimation resource algorithms, CIBERSORT, and single sample gene set enrichment analyses.

**Results:**

Consensus clustering classified the patients with BRCA into two subgroups with significantly different overall survival rates and immune microenvironments. Better prognosis was associated with high immune infiltration. The prognostic risk model, based on four LMAGs (*MED10*, *PLA2G2D*, *CYP4F11*, and *GPS2*), successfully stratified the patients into high- and low-risk groups in both the training and validation sets. High risk scores predicted poor prognosis and indicated low immune status. Subgroup analysis suggested that the risk model was an independent predictor of prognosis in BRCA.

**Conclusion:**

This study demonstrated, for the first time, that LMAG expression plays a crucial role in BRCA. The LMAG-based risk model successfully predicted the prognosis and indicated the immune microenvironment of patients with BRCA. Our study may provide inspiration for further research on BRCA pathomechanisms.

## Introduction

Breast cancer (BRCA) is the most common malignancy and the second leading cause of cancer-related death among women globally (DeSantis et al., 2019). Epidemiological studies have revealed that at least 268,000 patients are newly diagnosed with BRCA each year and 41,760 patients die. The incidence of BRCA has been steadily increasing (Siegel et al., 2019; Britt et al., 2020; Zhao et al., 2020). Currently, the therapeutic strategies for BRCA mainly include surgical resection, endocrine therapy, and combining surgery with various types of adjuvant therapies, including radiotherapy, chemotherapy, and immunotherapy (Waks and Winer, 2019; Zhao et al., 2020). The therapeutic objective is to improve the long-term survival and quality of life of patients with BRCA. The 5-year overall survival rate for early diagnosis of BRCA is >90%, which declines to 27% in stage IV patients (DeSantis et al., 2019). BRCA mainly includes three subtypes: ERBB2+, hormone receptor positive/ERBB2 negative (HR+/ERBB2-), and triple-negative. As the tumors are remarkably heterogeneous, fixed-treatment modes are not effective for all patients. Considering the significant tumor heterogeneity of BRCA, multivariable indicators are more significant than single biomarkers for prognosis prediction (Zhang et al., 2020), and risk models based on gene expression are a promising option (Yu et al., 2019).

The dysregulation of lipid metabolism plays a pivotal role in tumorigenesis and development, and increasing evidence indicates that lipid metabolism is essentially reprogrammed in tumors. This is regarded as a new hallmark of malignant tumors (Cheng et al., 2018; Maan et al., 2018; Visweswaran et al., 2020; Matsushita et al., 2021). Growing data suggest that lipid-metabolic reprogramming also plays an important role in the invasion and metastasis of malignant tumors (Luo et al., 2017). Targeting the lipid metabolism of tumor cells has been recognized as an attractive tumor treatment strategy (Liu et al., 2017; Visweswaran et al., 2020). Emerging evidence also indicates that aberrant lipid metabolism is involved in drug resistance during cancer treatment (Iwamoto et al., 2018; Cao, 2019). Previous studies have reported that risk signatures derived from lipid metabolism-associated genes (LMAGs) exhibit potent capability in predicting the prognosis of various tumors, including serous ovarian carcinomas (Zheng et al., 2020), clear cell renal cell carcinomas (Zheng et al., 2020), pancreatic cancer (Ye et al., 2021), lung adenocarcinomas (Li et al., 2020), and diffuse gliomas (Li et al., 2020). However, the prognostic value of LMAGs in patients with BRCA remains largely unknown.

The tumor microenvironment (TM) is a crucial regulator of malignancy (Wu et al., 2021). In particular, the tumor immune microenvironment, which reflects the immune status of the tumor tissues, performs critical functions, and has attracted increasing attention (Pitt et al., 2016; Locy et al., 2018; Lei et al., 2020). Immune cells in the immune microenvironment possess effective regulatory and destructive effects on tumor cells, and may have dual tumor-promoting and tumor-antagonizing roles (Lei et al., 2020). Recently, various studies have demonstrated that the immune microenvironment is crucial for the development and therapeutic efficacy of tumors (Lei et al., 2020). Meanwhile, increasing evidence indicates that dysregulation of the lipid metabolism greatly influences the immune microenvironment (Hao et al., 2019). However, the association between LMAGs and the tumor immune microenvironment remains obscure in BRCA.

Therefore, in this study, we explore the role of lipid metabolism in the oncogenesis and development of BRCA, using multiple bioinformatics methods. A novel prognostic risk model was constructed, based on LMAG expression levels, to evaluate the prognostic value of LMAGs in patients with BRCA. We also comprehensively analyzed differences in the immune microenvironments of patients with BRCA. In addition, we preliminarily elucidated the potential signaling pathways involved.

To the best of our knowledge, this is the first study to report the prognostic role of LMAGs in BRCA. The results of this study should provide inspiration for elucidating the molecular mechanisms of BRCA tumorigenesis and progression, promoting the development of personalized targeted therapy, and improving the prognosis of patients with BRCA.

## Materials and Methods

### Data Collection

**FIGURE 1:**
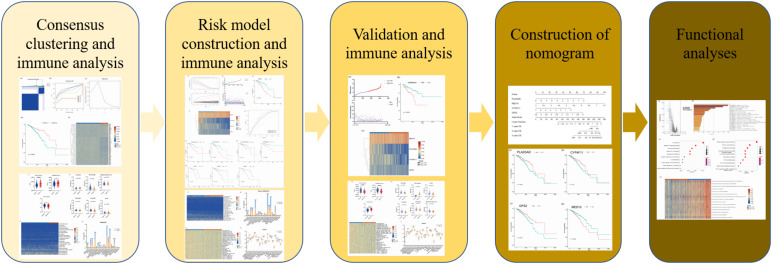
The scheme diagram of data analyzing.

The clinical data and gene expression matrices of enrolled patients were obtained from The Cancer Genome Atlas database^1^. In this study, 1076 BRCA samples were included, of which 760 samples were assigned to the training cohort and 316 to the validation cohort. The baseline data of all the samples are summarized in Table 1. A total of 776 LMAGs were collected from the Kyoto Encyclopedia of Genes and Genomes (KEGG) and Reactome databases, and 78 of these were identified as prognostic for BRCA, using univariable Cox regression analysis. And the process of data analysis is shown in Figure 1.

**TABLE 1:** Clinical characteristics of samples.

Variable	Training cohort (*N* = 760)	Validation cohort (*N* = 316)
	*N* (%)	*N* (%)
**Age**		
<58 years old	377 (49.6)	145 (45.9)
≥58 years old	383 (50.4)	171 (54.1)
**Metastasis**		
Yes	24 (3.2)	10 (3.2)
No	736 (96.8)	306 (96.8)
**Tumor stage**		
Stage 1	137 (18.0)	51 (16.1)
Stage 2	430 (56.6)	183 (57.9)
Stage 3	167 (22.0)	74 (23.4)
Stage 4	26 (3.4)	8 (2.5)
**Race**		
Asian	37 (4.9)	19 (6.0)
Black or African American	124 (16.3)	39 (12.3)
White	546 (71.8)	227 (71.8)
Unknown	53 (7.0)	31 (9.8)
**Radiotherapy**		
Yes	389 (51.2)	157 (49.7)
No	286 (37.6)	133 (42.1)
Unknown	85 (11.2)	26 (8.2)

### Consensus Clustering

According to the expression matrix of the 78 LMAGs, consensus clustering was performed using the R package “ConsensusClusterPlus” to divide the patients with BRCA in the training cohort into subgroups. Clustering was implemented on the grounds of partitioning around medoids, with Euclidean distances.

### Immune Analysis

The estimation of stromal and immune cells in malignant tumor tissues using expression data (ESTIMATE) method was applied to calculate the immune score, ESTIMATE score, and tumor purity of the patients, via the R package “estimate” (Yoshihara et al., 2013). Tumor immune estimation resource (TIMER) analysis^2^ was conducted to evaluate the abundance of six types of immune cells (neutrophils, CD4 T cells, macrophages, CD8 T cells, dendritic cells, and B cells) (Li et al., 2017). The CIBERSORT algorithm was employed to estimate the ratio of 22 types of infiltrating immune cells. Single sample gene set enrichment analysis (ssGSEA) was performed to assess the infiltration level of 28 types of immune cells using the “GSVA” R package.

### Construction and Validation of Risk Model Based on LMAGs

Based on univariable regression analysis, the least absolute shrinkage and selection operator algorithm was applied using the R package “glmnet” to select candidate genes for constructing the risk model. The genes included in the risk model were determined using multivariate regression analysis. Which were listed in Table 2. Each patient in both the training and validation cohorts was assigned a risk score, according to the following formula: risk score = (−0.2141 × *PLA2G2D* expression) + (0.52944 × *MED10* expression) + (−0.1887 × *CYP4F11* expression) + (−0.4069 × *GPS2* expression). The patients were categorized into low- and high-risk groups, with the median of the risk score regarded as the cutoff. Kaplan–Meier analysis was employed to estimate the difference in overall survival between the categorized patients via the R package “survival.” The prognostic capability of the risk model was validated using time-dependent receiver operating characteristic (ROC) analysis with the R package “survivalROC.”

**TABLE 2:** Features of the genes used for constructing the risk model.

Gene name	Full name	Category	Function
MED10	Mediator Complex Subunit 10	Protein Coding	Component of the Mediator complex, a coactivator involved in regulating the transcription of nearly all RNA polymerase II-dependent genes
PLA2G2D	Phospholipase A2 Group IID	Protein Coding	Secretory calcium-dependent phospholipase A2 that primarily targets extracellular lipids, exerting anti-inflammatory, and immunosuppressive effects
CYP4F11	Cytochrome P450 Family 4 Subfamily F Member 11	Protein Coding	A cytochrome P450 monooxygenase involved in the metabolism of various endogenous substrates, including fatty acids and their oxygenated derivatives
GPS2	G Protein Pathway Suppressor 2	Protein Coding	Key regulator of inflammation, lipid metabolism, and mitochondrion homeostasis that acts by inhibiting the activity of the ubiquitin-conjugating enzyme UBE2N/Ubc13, thereby inhibiting ‘Lys-63’-linked ubiquitination

### Evaluation of Risk Model Independence

Univariate and multivariate Cox regression analyses were performed to estimate whether the risk score was an independent predictor of BRCA prognosis. A subgroup analysis was conducted to confirm the independence of the risk model. The patients with BRCA in the training cohort were regrouped into new subgroups based on different clinical characteristics, and the patients in each subgroup were stratified into high- and low-risk groups, based on the median risk score.

### Functional Analysis

Differential expression analysis was conducted using the “limma” R package to identify differentially expressed genes (DEGs) for subsequent analyses. Gene ontology (GO) and KEGG analyses were performed to enrich the DEGs into associated pathways using the “clusterProfiler” R package. Enrichment analysis was also performed using the web tool “metascape”^3^. Moreover, the activity of each GO term in each patient with BRCA was evaluated via gene set variation analysis (GSVA) using the “GSVA” R package.

### Statistical Analysis

Data analyses were mainly completed using R (version 4.0) and GraphPad Prism (version 8.0), and visualization also employed TBtools (Chen et al., 2020). Discontinuous data are presented as number (percentage), and continuous data are displayed as mean ± standard deviation. Differences between two groups were calculated using Student’s *t*-tests, while those among more than two groups were calculated using one-way ANOVA. Statistical significance was defined as *p* < 0.05.

## Results

### Consensus Clustering for LMAGs Correlated With Prognosis and Immune Microenvironment in BRCA

**FIGURE 2:**
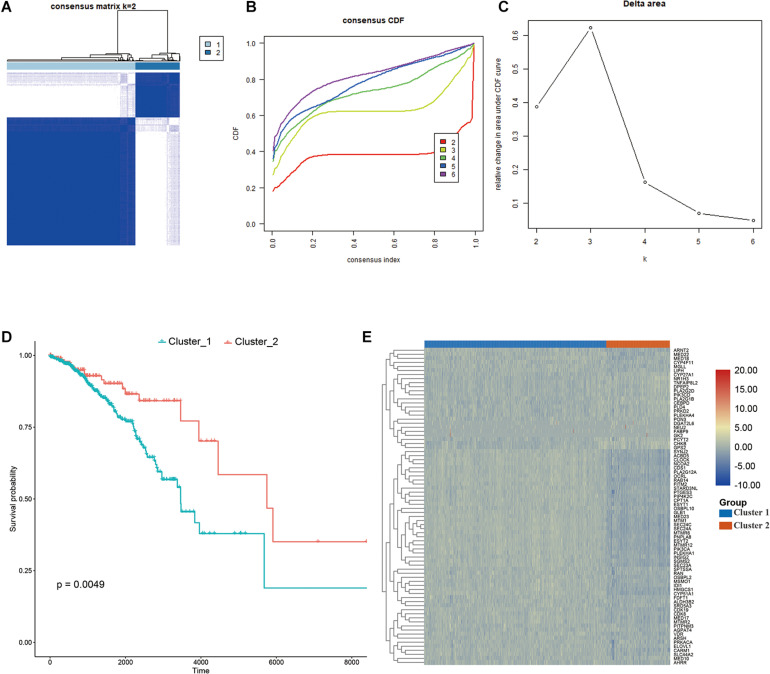
Consensus clustering. **(A–C)** The clustering demonstrating the best stability with *K* = 2, **(D)** Kaplan–Meier analysis curve, **(E)** heatmap illustrating the different expressional pattern of the prognostic lipid metabolism associated genes in the two clusters.

**FIGURE 3:**
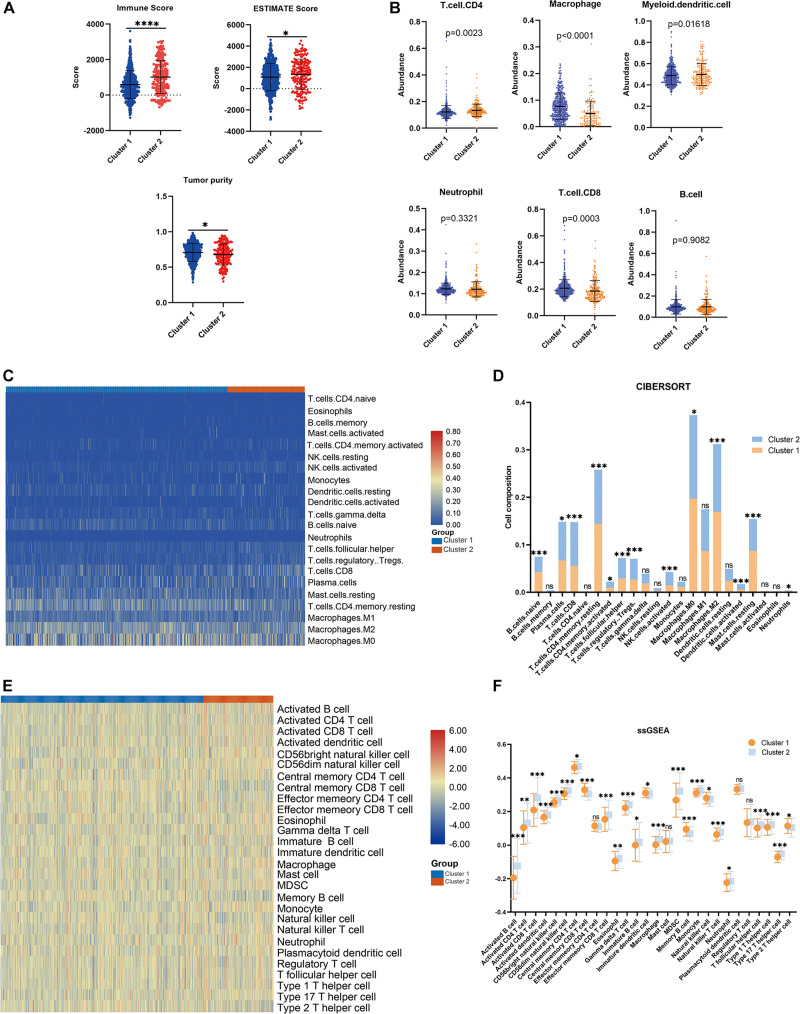
Immune analyses in the clustered subgroups. **(A)** ESTIMATE algorithm, **(B)** TIMER algorithm, **(C,D)** CIBERSORT analysis, and ssGSEA analysis **(E,F)**. *p < 0.05, **p < 0.01, ***p < 0.001, ****p < 0.0001.

Consensus clustering was conducted to cluster the patients with BRCA in the training cohort into subgroups. The results showed that consensus clustering was the most stable when *K* = 2 (Figures 2A–C and Supplementary Figure 1). Therefore, the patients with BRCA were divided into two groups, with 564 patients in Cluster 1 and 196 patients in Cluster 2. Survival analysis indicated that the overall survival of the two clusters differed, and that patients in Cluster 2 had a significantly better prognosis (Figure 2D). Heatmap visualization showed that the prognostic LMAG expression of the patients with BRCA also differed significantly between the two clusters (Figure 2E). The ESTIMATE assessment suggested that the patients in Cluster 2 had higher immune scores and ESTIMATE scores and lower tumor purity (Figure 3A). In addition, TIMER analysis demonstrated a statistical difference between the two clusters regarding the abundance of infiltrating immune cells. The abundance of CD4 T cells and myeloid dendritic cells was lower in Cluster 1 than in Cluster 2, whereas the opposite trend was observed in macrophage cells and CD8 T cells (Figure 3B). Meanwhile, the CIBERSORT algorithm was used to calculate the infiltration level of the immune cells (Figure 3C), and quantitative analysis suggested a significant difference between the two clusters (Figure 3D). Finally, ssGSEA was performed to assess the immune status of the patients with BRCA, and the results are illustrated in a heatmap (Figure 3E), which shows that the patients in Cluster 2 had a relatively high immune status compared with those in Cluster 1. Statistical analysis confirmed the ssGSEA results (Figure 3F). These findings indicate that LMAG expression is associated with the prognosis and immune microenvironment of patients with BRCA.

### Construction of an LMAG-Based Risk Model Using the Training Cohort

**FIGURE 4:**
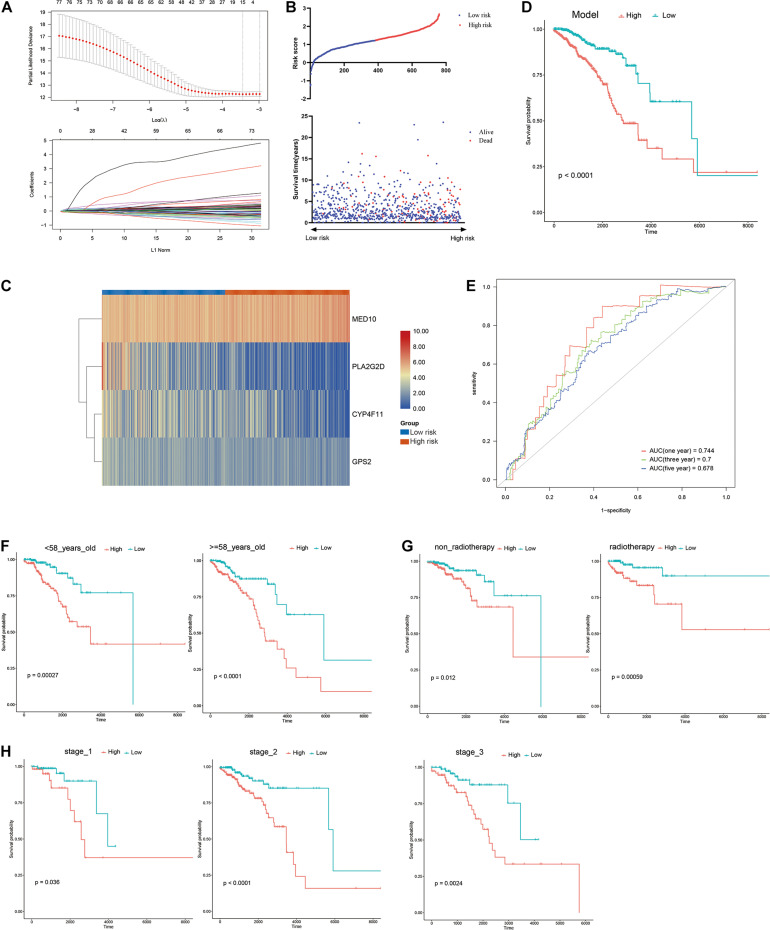
Construction of risk model. **(A)** LASSO analysis revealing the minimal lambda, **(B)** survival status and risk score, **(C)** heatmap visualizing the expression pattern of the four candidate LMAGs, **(D)** survival curve illustrating the overall survival of the BRCA patients, **(E)** time-dependent ROC curve, **(F–H)** subgroup analyses suggesting the independence of the risk model regarding age, radiotherapy, and tumor stage.

A risk model based on LMAGs was established to evaluate the prognostic value of LMAGs in BRCA. Based on univariate Cox analysis, least absolute shrinkage and selection operator analysis screened out 15 genes for subsequent analysis (Figure 4A). Of these, multivariate Cox analysis identified four genes to include in the constructed risk model, *MED10*, *PLA2G2D*, *CYP4F11*, and *GPS2*, all of which can independently predict the prognosis of patients with BRCA (Supplementary Figure 2). According to the median risk score, the patients were categorized into high- and low-risk groups. The risk score and survival status distributions of the patients are depicted in Figure 4B. A heatmap visualizing the expression pattern of the genes used in the risk model suggested that patients in the high-risk group tended to express risk genes with a hazard ratio > 1, including *MED10*, while patients in the low-risk group tended to express protective genes with a hazard ratio < 1, including *PLA2G2D*, *CYP4F11*, and *GPS2* (Figure 4C). The Kaplan–Meier survival curve indicated that the patients in the low-risk group had significantly better overall survival (Figure 4D). With respect to model diagnosis, the areas under the curve of the time-dependent ROC curves were 0.744 for one-year survival, 0.700 for three-year survival, and 0.678 for five-year survival, respectively, suggesting the acceptable stability of the risk model (Figure 4E). These results indicate that the LMAG-based risk model could accurately predict the prognosis of patients with BRCA.

### The Risk Model Is an Independent Indicator for BRCA Prognosis

Univariable/multivariable Cox regression and subgroup analyses were conducted to verify the independence of the LMAG-based risk model. Univariate Cox regression analysis revealed that the risk score could predict the prognosis of patients with BRCA independently (*p* = 2.47E−10) (Supplementary Table 1). In the multivariable regression analysis, the risk score remained statistically significant for BRCA survival (*p* = 1.42E−06) (Supplementary Table 2). Additionally, the patients with BRCA in the training cohort were regrouped into subgroups based on age (<58 and ≥58 years old), tumor stage (stage 1, stage 2, and stage 3), and radiotherapy application. Regardless of the subgroups, the LMAG-based risk model stratified the patients with BRCA into the high- and low-risk groups, and the patients in the low-risk group still showed significantly longer survival (Figures 4F–H). These results indicate the excellent independence of the risk model.

### The Risk Model Was Associated With Immune Microenvironment in the Training Cohort

**FIGURE 5:**
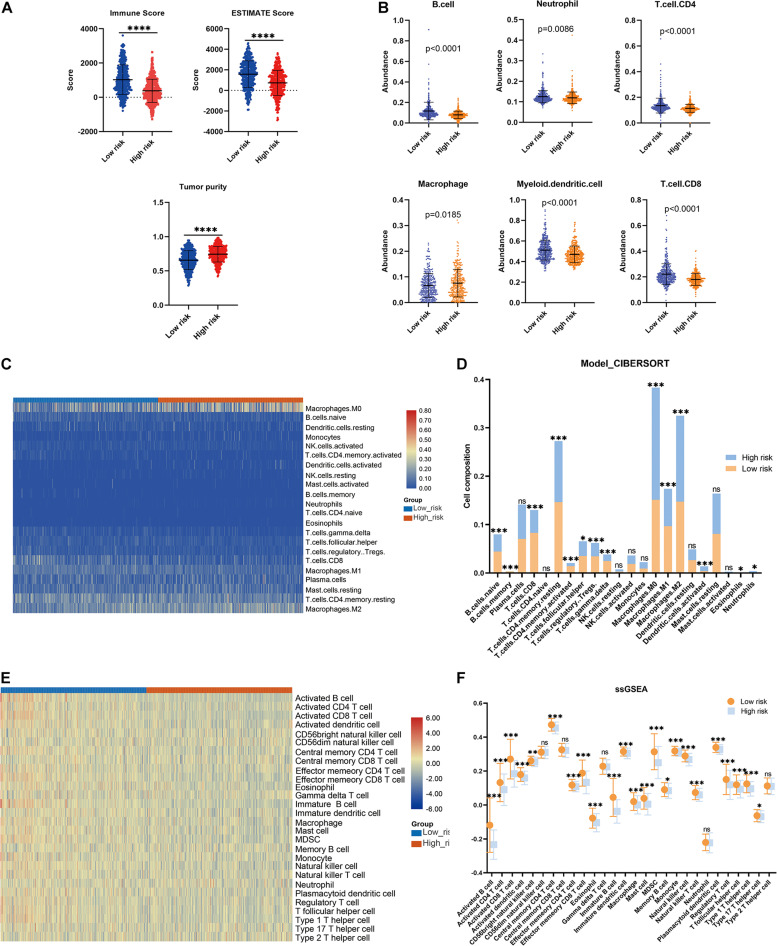
Immune microenvironment landscape in the training cohort. **(A)** ESTIMATE algorithm, **(B)** TIMER algorithm, **(C,D)** CIBERSORT analysis, and ssGSEA analysis **(E,F)**. *p < 0.05, **p < 0.01, ***p < 0.001, ****p < 0.0001.

The association between the risk model and the immune microenvironment in BRCA was assessed using multiple immune analyses. The ESTIMATE results revealed that the patients with BRCA in the high-risk group had significantly lower immune scores and ESTIMATE scores, and higher tumor purity, than those in the low-risk group (Figure 5A). The TIMER algorithm indicated that the abundances of B cells (*p* < 0.0001), neutrophils (*p* = 0.0086), CD4 T cells (*p* < 0.0001), myeloid dendritic cells (*p* < 0.0001), and CD8 T cells (*p* < 0.0001) in the low-risk group were statistically higher than those in the high-risk group, while the opposite was observed for macrophages (Figure 5B). The results of the CIBERSORT immune-infiltration analysis are depicted in a heatmap (Figure 5C), and the corresponding statistical analysis suggested significant differences in most immune-infiltrating cells (Figure 5D). Moreover, ssGSEA revealed that the patients with BRCA in the low-risk group had a relatively high immune status compared with those in the high-risk group (Figure 5E). Meanwhile, quantitative analysis confirmed higher ssGSEA scores in the low-risk group than in the high-risk group (Figure 5F). The above findings demonstrate that the LMAG-based risk model was related to the immune microenvironment in BRCA.

### The Risk Model Correlated With Prognosis and Immune Microenvironment in the Validation Cohort

**FIGURE 6:**
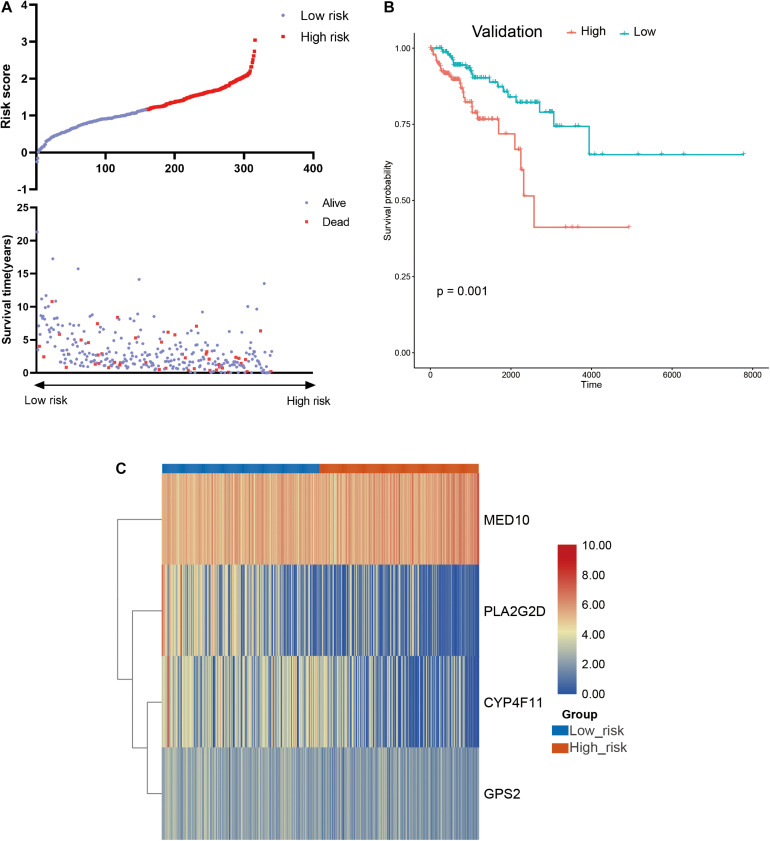
Verification of the risk model in the validation cohort. **(A)** Survival status and risk score in the validation set, **(B)** survival curve showing the survival of the patients in the validation cohort, **(C)** heatmap illustrating the expression of candidate LMAGs in the validation cohort.

**FIGURE 7:**
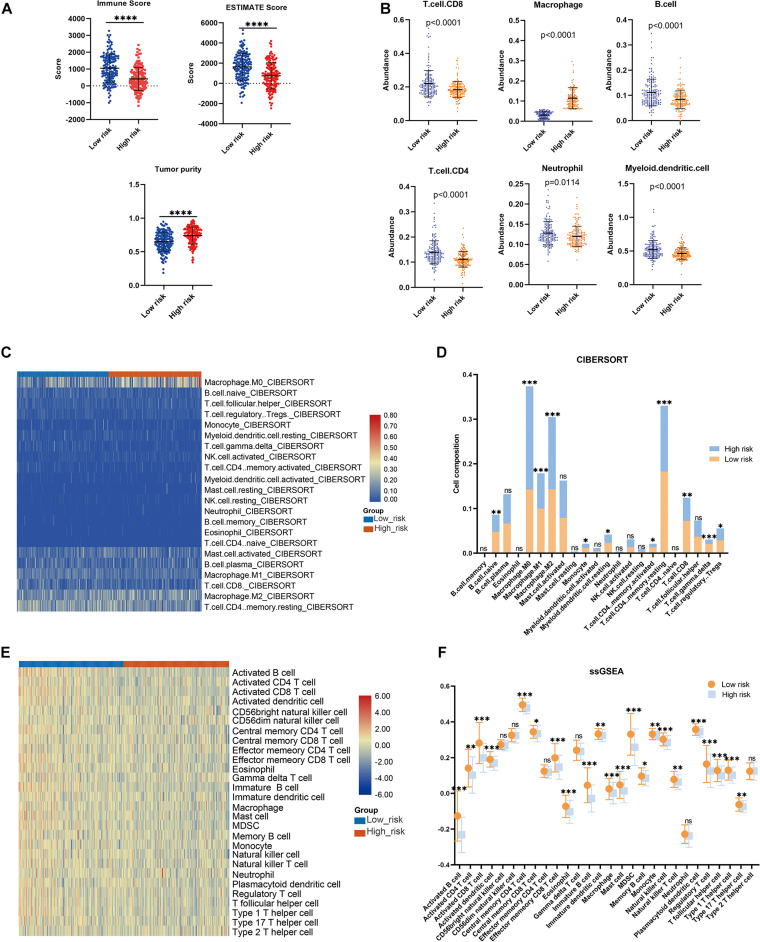
Association between the risk model and immune microenvironment in the validation cohort. **(A)** ESTIMATE algorithm, **(B)** TIMER algorithm, **(C,D)** CIBERSORT analysis, and ssGSEA analysis **(E,F)**. *p < 0.05, **p < 0.01, ***p < 0.001, ****p < 0.0001.

To further confirm the practicability and reliability of the LMAG-based risk model, it was verified using the validation cohort. According to the median risk score, the patients in the validation cohort were stratified into high- and low-risk groups. The survival status and risk score distributions are illustrated in Figure 6A. As expected, the overall prognosis of the two groups differed significantly, and the patients in the low-risk group showed longer survival (*p* = 0.001) (Figure 6B). The expressions of the four genes included in the risk model are shown in a heatmap (Figure 6C). Patients in the low-risk group showed a tendency to express protective genes (*PLA2G2D*, *CYP4F11*, and *GPS2*), whereas patients in the high-risk group tended to express the risk gene *MED10*. Furthermore, the relationship between the risk score and the BRCA immune microenvironment was confirmed in the validation cohort. In the ESTIMATE analysis, a low risk score was significantly associated with high immune and ESTIMATE scores, and low tumor purity (Figure 7A). Regarding the TIMER analysis, the abundance of all six immune cells was statistically different between the two groups (Figure 7B). Except for macrophages (*p* < 0.0001), the abundances of all the immune cells were significantly higher in the low-risk group than in the high-risk group. The heatmap visualization and corresponding statistical analysis of the CIBERSORT algorithm are depicted in Figures 7C,D, respectively. In the ssGSEA, relatively high immune status was observed in the low-risk group compared to the high-risk group (Figure 7E), and quantitative analysis identified statistical differences in most immune cells between the two groups (Figure 7F). These results suggest that the LMAG-based risk model is related to prognosis and the immune microenvironment in BRCA.

### Establishment of a Risk Model-Based Nomogram in the Training Cohort

**FIGURE 8:**
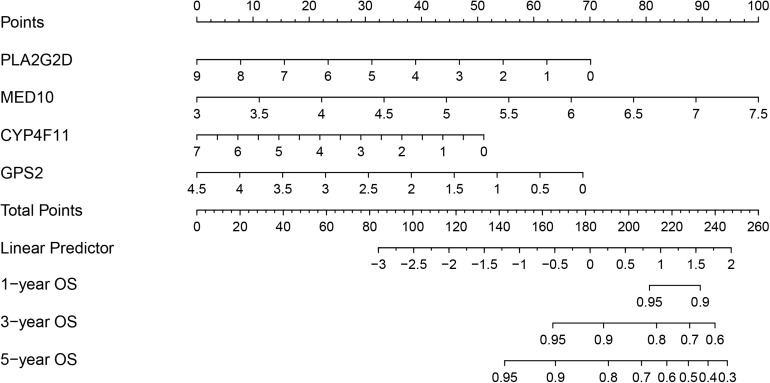
Nomogram based on the prognosis-associated lipid metabolism-associated genes.

To develop a strategy to quantitatively predict the probability of survival in BRCA, a prognostic nomogram was constructed, based on the four genes from the risk model, via Cox proportional hazards analysis (Figure 8). As depicted in the nomogram, each prognostic gene in the risk model was endowed with a specific score and a corresponding expression value, and the total score was obtained by summing the scores of all the variables. Correspondingly, the survival probability of patients with BRCA can be calculated according to the total score. The one-year, three-year, and overall survival rates of the patients with BRCA were predicted using the nomogram.

### Immune-Related Signaling Pathways May Mediate the Role of LMAGs in BRCA

**FIGURE 9:**
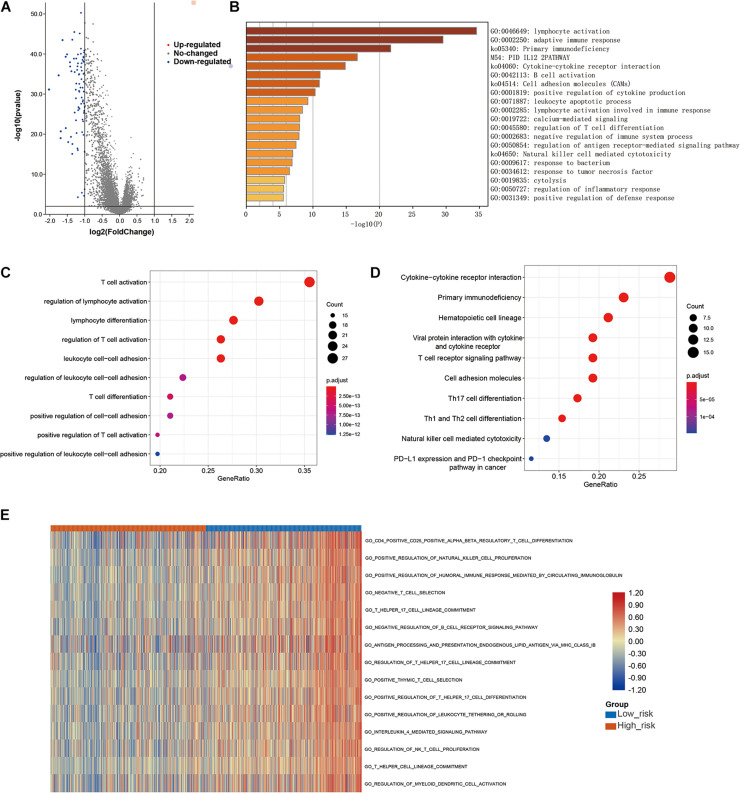
Functional analyses. **(A)** Volcano plot depicting the differentially expressed genes (DEGs) between the two groups, **(B,C)** bubble plot derived from gene ontology (GO) analysis suggesting that the DEGs were mainly enriched in immune-associated biological processes, **(D)** bubble diagram derived from Kyoto Encyclopedia of Genes and Genomes (KEGG) analysis showing that the DEGs were enriched in immune-associated signaling pathways, **(E)** GSVA plots visualizing the result of GSVA analysis.

Finally, to further explore the molecular mechanisms associated with the roles of LMAGs in BRCA, functional analyses were conducted. Eighty DEGs were identified between the high-risk and low-risk groups; the volcano plot is shown in Figure 9A. It is worth noting that all of the DEGs were downregulated in the high-risk group compared with the low-risk group. Enrichment analyses revealed that the DEGs were mainly enriched in immune-related signaling pathways (Figures 9B–D), suggesting that immunity may mediate the significance of LMAGs in the prognosis of patients with BRCA. GO analysis indicated that the DEGs were mainly enriched in biological processes associated with immune cell activation, differentiation, and adhesion (Figure 9C), although they were also enriched in some crucial molecular functions and cellular components (Supplementary Figure 3). KEGG analysis demonstrated that the DEGs were enriched in signaling pathways related to immune cell differentiation (Figure 9D). Furthermore, GSVA was performed to calculate the activity of the GO terms in each patient with BRCA and the results, visualized in a heatmap (Figure 9E), suggest that the activities of immune-related pathways were relatively downregulated in the high-risk group. These findings indicate that immune-related signaling pathways may mediate the role of LMAGs in BRCA.

## Discussion

As one of the deadliest malignancies in women, BRCA has always been a serious public health issue and imposes a huge burden on humankind worldwide (Harbeck and Gnant, 2017; Batalha et al., 2021). Although the prognosis has improved significantly with the development of medical technology, the 5-year overall survival of patients with advanced BRCA is still far from satisfactory (Harbeck and Gnant, 2017; Tian et al., 2020). Constructing a risk model is an innovative and applicable way to predict a patient’s prognosis, and it provides a valuable supplement for the TNM stage system in patient risk stratification. Considering the significance of lipid metabolism in BRCA biology, we developed a prognostic model based on four LMAGs and estimated the effect of LMAGs on the immune microenvironment of patients with BRCA. The results suggest that the LMAG-based risk model possesses potent predictive capacity in the prognosis of patients with BRCA and can indicate the tumor immune microenvironment. Additionally, functional analyses showed that immune-associated signaling pathways mediated the role of LMAGs in BRCA. The results of this work could provide a novel perspective for future BRCA research, optimize risk stratification, promote the development of targeted therapy, and help to improve the prognosis of patients with BRCA.

It has been previously reported that the lipid metabolite 27OHC was a potential risk biomarker for BRCA and can act as a mitogen (Nazih and Bard, 2020). This means that abnormal lipid metabolism plays a pivotal role in the pathogenesis of BRCA. Immune abnormality is also an important risk factor for BRCA. The crosstalk between the immune system and cancer cells will change dynamically during the development and treatment of cancer (King et al., 2017).

LMAGs expression predicted the prognosis and was associated with the immune microenvironment of patients with BRCA. Consensus clustering is an acknowledged unsupervised clustering method that can identify new molecular subtypes according to different omics datasets (Wilkerson and Hayes, 2010). In this study, consensus clustering successfully classified the patients with BRCA into two clusters, according to the expression level of prognostic LMAGs. The patients in Cluster 2 had a favorable prognosis, suggesting that LMAG expression is valuable in the prediction of BRCA prognosis. Although further functional analyses were not performed to clarify the inherent associations and molecular cascades of the two newly identified molecular subgroups, the consensus clustering results provide a novel perspective for further research on BRCA.

As the two molecular subtypes are an innovative classification of BRCA, they provide a supplement or substitution for the existing categorization system. It should be noted that the ratio between the two groups was approximately three to one, which was not equal, but is acceptable. In addition, we found statistically significant differences in immune microenvironment between the two subgroups. In particular, better overall survival was correlated with higher immune scores, lower tumor purity, and relatively high immune status, which is consistent with a previous study (Qi et al., 2020). High tumor purity implies shorter survival time and such tumors are more likely to be diagnosed as malignant. Considering that the clustering was conducted based on an LMAG expression matrix, we could reasonably infer that LMAG expression was correlated with the immune microenvironment of patients with BRCA.

Immune cell infiltration into the TM reflects the immune landscape in tumors (Ho et al., 2020). Suppressing anti-tumor immune response is the main mechanism through which cancer cells evade supervision and destruction by the immune system (De Cicco et al., 2020). The functional roles of the various immune cells differ. Usually, CD8 T cells, CD4 T cells, dendritic cells, B cells, and NK cells exert anti-tumor functions, regulatory T cells play a pro-tumor role, and macrophages and monocytes play equivocal roles in the progression of tumor progression (Chimal-Ramírez et al., 2013; Zhang and Zhang, 2020; Batalha et al., 2021). Results of TIMER in clustered and risk model-stratified subgroups are not completely consistent in this study, especially CD8 T cell, which is worth further investigation. We constructed a risk model to estimate and validate the role of LMAGs in the immune microenvironment of BRCA. The established LMAG-based risk model stratified the patients into groups with different prognoses that were correlated with immune infiltration in both the training and validation cohorts. Moreover, model diagnosis using ROC analysis indicated that the risk model is a reliable indicator of prognosis. Overall, these results reveal that the LMAG-based risk model is a well-developed reference for predicting the prognosis of patients with BRCA, and that it is closely related to the tumor immune microenvironment in BRCA.

The constructed risk model in this study was not influenced by other clinical parameters, such as age. In addition to stability, independence is a critical index for an effective prognostic risk model. In this study, the prognosis was correlated with the risk score, and univariable and multivariate regression analyses indicated that the risk model was an independent indicator of BRCA prognosis. To further explore the possibility of interference resulting from other indexes, subgroup analysis was performed to confirm the prognosis-predicting power of the risk model. The results suggest that the risk score remains effective in predicting BRCA prognosis even when the patients are regrouped according to clinical parameters. Unfortunately, subgroup analysis of metastasis could not be conducted, as this factor was limited by sample size. The LMAG-based risk model was independently predictive of prognosis in BRCA.

All the genes in our constructed risk model were related to tumors prognosis. Risk models are an applicable method for developing prognostic molecular biomarkers and have been constructed for a variety of tumors, showing powerful predictive ability (Chen et al., 2021; Lv et al., 2021). Generally, the patients were scored according to candidate-gene expression values and coefficients, and lower scores indicated a favorable prognosis. In the present study, four candidate LMAGs, including one risk gene and three protective genes that have all been demonstrated to be involved in the progression and prognosis of tumors, were selected for the construction of the risk model.

*MED10* encodes the mediator of RNA polymerase II transcription subunit 10 (MED10), and previous studies have suggested that *MED10* expression is a risk factor for multiple types of tumors (Xu et al., 2018; Sahar et al., 2019; Wang et al., 2021). Xu et al. (2018) and Wang et al. (2021) demonstrated that *MED10* is a critical prognostic gene in glioblastoma and hepatocellular carcinoma, respectively. The protein level of *MED10* has also been shown to be upregulated in uterine leiomyoma compared to that in the adjacent myometrium (Sahar et al., 2019). In line with the above reports, *MED10* was shown to be detrimental for the prognosis of patients with BRCA, and tended to be expressed in the high-risk group in this study. Phospholipase A2 group IID (PLA2G2D), encoded by *PLA2G2D*, is an immunosuppressor involved in the conversion of lipid balance to an anti-inflammation status, which can play a detrimental or beneficial role, depending on inflammatory and tumorous context (Miki et al., 2016). A previous study revealed that the expression of *PLA2G2D* was reduced 23-fold in colorectal adenocarcinomas compared to normal tissue (Mounier et al., 2008; Xiong et al., 2021). Xiong et al. (2021) reported that *PLA2G2D* is a prognosis-predicting gene in head and neck squamous cell carcinoma. The cytochrome P450 (CYP) superfamily are the most important microsomal mixed functional oxidases. *CYP4F11* encodes a member of this superfamily, cytochrome P450, family 4, subfamily F, polypeptide 11 (CYP4F11), which catalyzes the formation of stearoyl CoA desaturase inhibitors (Theodoropoulos et al., 2016). The expression of *CYP4F11* has been significantly and independently correlated with survival in esophageal squamous cell carcinoma and colorectal cancer (Alnabulsi et al., 2017; Wu et al., 2018). It is especially worth noting that the gene expression level of *CYP4F11* in BRCA is significantly higher than that in adjacent tissues (Bandala et al., 2012). Finally, *GPS2* encodes G protein suppressor 2 (GPS2), which was identified as a constituent of the silencing mediator of retinoic acid and thyroid hormone receptor corepressor complexes (Cheng and Kao, 2009). *GPS2* has been shown to play a tumor-suppressive role in liposarcoma (Huang et al., 2016) and BRCA (Cheng and Kao, 2009; Chan et al., 2020), and the loss of GPS2 facilitated tumor growth and the proliferation of cancer cells (Chan et al., 2020). These findings indicate that the aberrant expression of the candidate LMAGs is involved in the progression and prognosis of multiple types of tumors, including BRCA; therefore, it was reasonable to integrate them to establish a risk model for risk stratification and prognosis prediction in BRCA.

Immune-associated signaling cascades mediate the significance of LMAGs in BRCA. The GO and KEGG analyses suggested that the DEGs between the high- and low-risk groups were mainly enriched in immune-associated pathways. More specifically, GSVA revealed that patients in the low-risk group had a relatively high immune status. Considering the overall survival of the patients, it was reasonable to deduce that a favorable prognosis correlated with high immune cell infiltration. The patients were classified based on their LMAG expression values; thus, we concluded that the risk model predicted prognosis and indicated the immune microenvironment.

In recent years, increasing numbers of pre-clinical and clinical studies have highlighted the crucial role of metabolism in the clinical and immune responses of patients with cancer (Bleve et al., 2020). Emerging evidence has revealed an intricate interplay between lipid metabolism and immune cell responses in tumors (Chen and Sui, 2021; Xiang and Miao, 2021). The detailed mechanisms can be interpreted in multiple dimensions. As mentioned previously, lipid-metabolic reprogramming is a new hallmark of malignant cancers. It is mostly altered to meet the requirements of nutrients for cellular proliferation, and could impact the state and functions of infiltrating immune cells (Liu W. et al., 2021; Qin and Chen, 2021; Xiang and Miao, 2021). Macrophages can be reprogrammed to promote tumor progression via increased cholesterol efflux (Goossens et al., 2019; Matsushita et al., 2021). Exogenous and endogenous lipid metabolism exerts different functions on T cells (Matsushita et al., 2021). The anti-tumor role of T cells may be enhanced by cholesterol; however, its role is negatively regulated by liver X receptors in the oxysterol-abundant TM (Matsushita et al., 2021). A recent study demonstrated that lipid metabolism reprograming, triggered by the unbalanced lipid metabolism in senescent T cells, prevented the senescence of effector T cells (Liu X. et al., 2021) and enhanced the functional specialization of regulatory T cells in cancers (Lim et al., 2021). Lipid accumulation in the dendritic cells damaged their anti-tumor functions, as the affected cells could not effectively present tumor-associated antigens (Herber et al., 2010). High levels of lipids in the TM can stimulate the generation of myeloid-derived suppressor cells, resulting in significant metabolic and functional rewiring (Al-Khami et al., 2017). Moreover, lipid metabolism is one of the most essential energy sources for various cells in the TM. Competition for lipid-metabolic nutrients between proliferative tumor cells and immune-infiltrating cells greatly influences their metabolic status and drastically modifies their functional phenotypes (Chang et al., 2015; O’Neill et al., 2016; Bleve et al., 2020). Thus, it was not surprising that LMAG expression was associated with the immune microenvironment in BRCA, or that a better prognosis was associated with high immune scores and a high abundance of immune cells. Additionally, some LMAGs are also responsible for immune regulation, exerting immune-suppressing or immune-promoting functions. In particular, *PLA2G2D*, one of the genes used to construct the risk model in this study, is abundantly expressed in the dendritic cells of lymphoid tissues and has anti-inflammatory and immunosuppressive functions (Miki et al., 2016). The expression of these dual-role LMAGs modifies the activity of immune-related pathways, leading to alterations in immune cell function and infiltration. These findings suggest that immune-related pathways mediate the effects of LMAG expression on the immune microenvironment and prognosis of BRCA.

The results of this study demonstrate that the LMAG-based risk model was connected with the tumor immune microenvironment and with prognosis in BRCA. To the best of our knowledge, this is the first study to report the function of LMAGs in the TM and prognosis of BRCA. Our findings provide innovative insights and could be used as a reference for targeted therapy in patients with BRCA.

### Strengths and Shortcomings

Several risk-stratification models have been constructed to predict the prognosis of patients with BRCA. However, our study has some unique assets. Above all, this study constructed a prognostic model based on lipid metabolism, which fills the gap of a LMAG-related risk model for predicting BRCA prognosis. Moreover, we not only focused on the predictive performance of LMAGs but also explored the effect of LMAG expression on the tumor immune microenvironment of patients with BRCA. In addition, the molecular mechanisms accounting for the prognostic role of LMAGs in BRCA were preliminarily elucidated and we constructed a nomogram to quantify the influence of each candidate LMAG on the survival of patients with BRCA. We acknowledge that there are some inevitable limitations to this work. First, our conclusion was drawn based on open datasets, but not on the sequenced data of our cohorts. Second, the results of this study were not further validated using clinical samples or laboratory experiments.

## Conclusion

In summary, this study comprehensively evaluates the role of LMAGs in the prognosis and immune microenvironment of patients with BRCA and explores the molecular mechanisms involved. The LMAG-based risk model that we constructed successfully predicted the overall survival of patients and indicated the tumor immune microenvironment in BRCA. In addition, our results suggest that immune-associated signaling pathways might mediate the functions of LMAGs in BRCA. Our work provides an innovative perspective for future BRCA research and the development of targeted therapy strategies for patients with BRCA. Further studies are needed to validate the clinical prognostic value of the LMAG-based risk model and to explore the underlying mechanisms.

## Data Availability Statement

The datasets presented in this study can be found in online repositories. The names of the repository/repositories and accession number(s) can be found in the article/Supplementary Material.

## Author Contributions

YH conceived the original ideas of this manuscript, reviewed the finished manuscript, and executed supervision throughout the process. ZY and SZ executed the data collection and data analysis. ZY, SZ, ZN, and ZX prepared the manuscript. ZY prepared the tables and figures. All authors contributed to the article and approved the submitted version.

## Conflict of Interest

The authors declare that the research was conducted in the absence of any commercial or financial relationships that could be construed as a potential conflict of interest.

## Footnotes

1 https://portal.gdc.cancer.gov/

2 https://cistrome.shinyapps.io/timer/

3 https://metascape.org
