# Supplementary material for: A Novel Risk Model Based on Lipid Metabolism-Associated Genes Predicts Prognosis and Indicates Immune Microenvironment in Breast Cancer
Source: Front Cell Dev Biol. 2021 Jun 14;9:691676. doi: 10.3389/fcell.2021.691676 (PMC8236894; doi:10.3389/fcell.2021.691676)
Supplement: Supplementary file 1 [file Data_Sheet_1.docx]

**Supplementary table 1**. Univariate regression analysis for independence

| Variates | coef | HR | HR.95L | HR.95H | pvalue |
| --- | --- | --- | --- | --- | --- |
| Risk score | 1.173999 | 3.234904 | 2.248859 | 4.653295 | 2.47E-10 |
| Age | 0.014382 | 1.014486 | 0.999572 | 1.029621 | 0.056999 |
| Tumor stage | 0.315652 | 1.371154 | 1.123996 | 1.672659 | 0.001854 |

Coef: coefficient; HR: Hazard Ratio

**Table S2**. Multivariate regression analysis for independence

| Variates | coef | HR | HR.95L | HR.95H | P value |
| --- | --- | --- | --- | --- | --- |
| Risk score | 0.898603001 | 2.456169 | 1.704733 | 3.538834 | 1.42E-06 |
| Age | 0.022278396 | 1.022528 | 1.007579 | 1.037700 | 0.003030 |
| Tumor stage | 0.390756218 | 1.478098 | 1.193851 | 1.830023 | 0.000336 |

Coef: coefficient; HR: Hazard Ratio


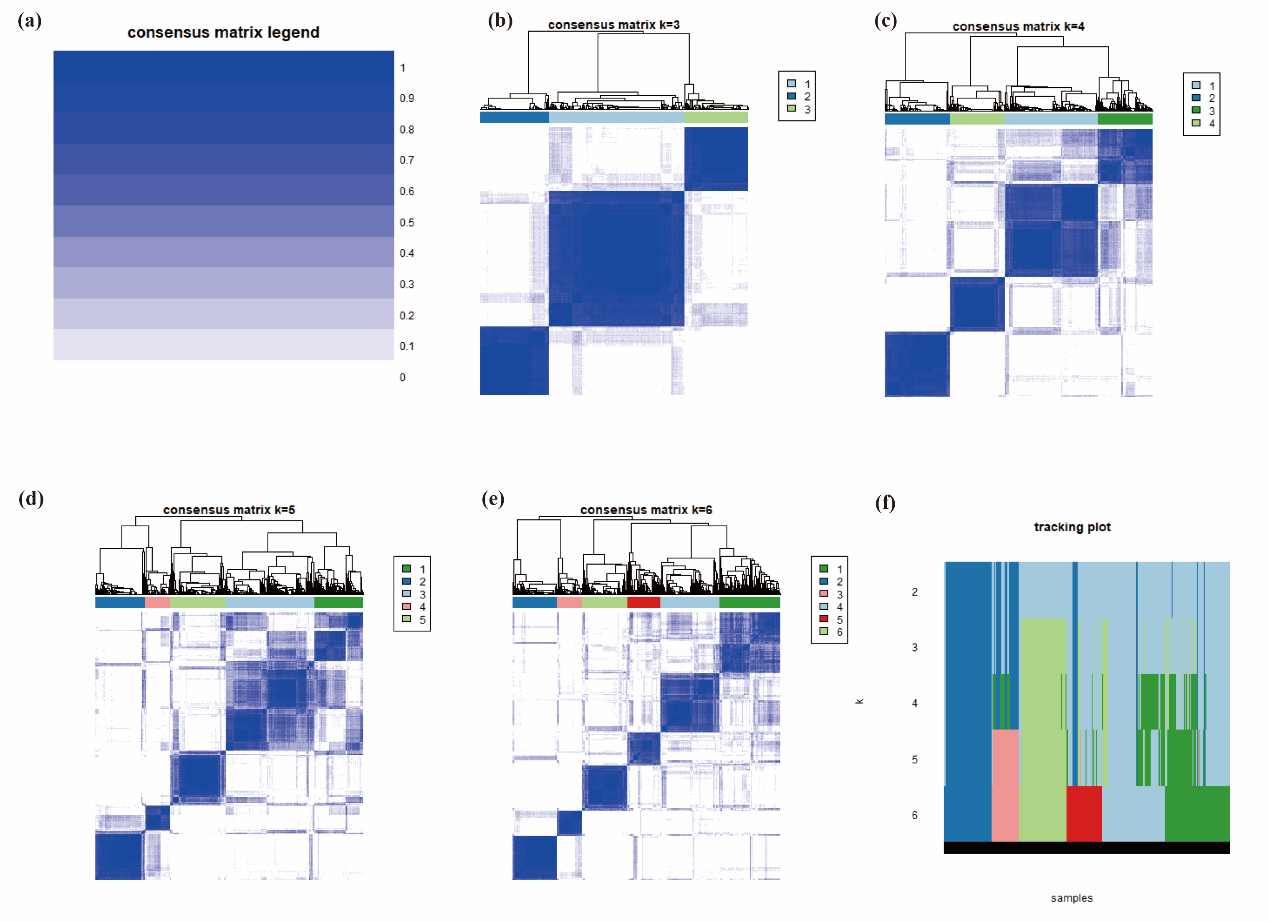


**Supplemental figure 1**. Consensus clustering of breast cancer patients.


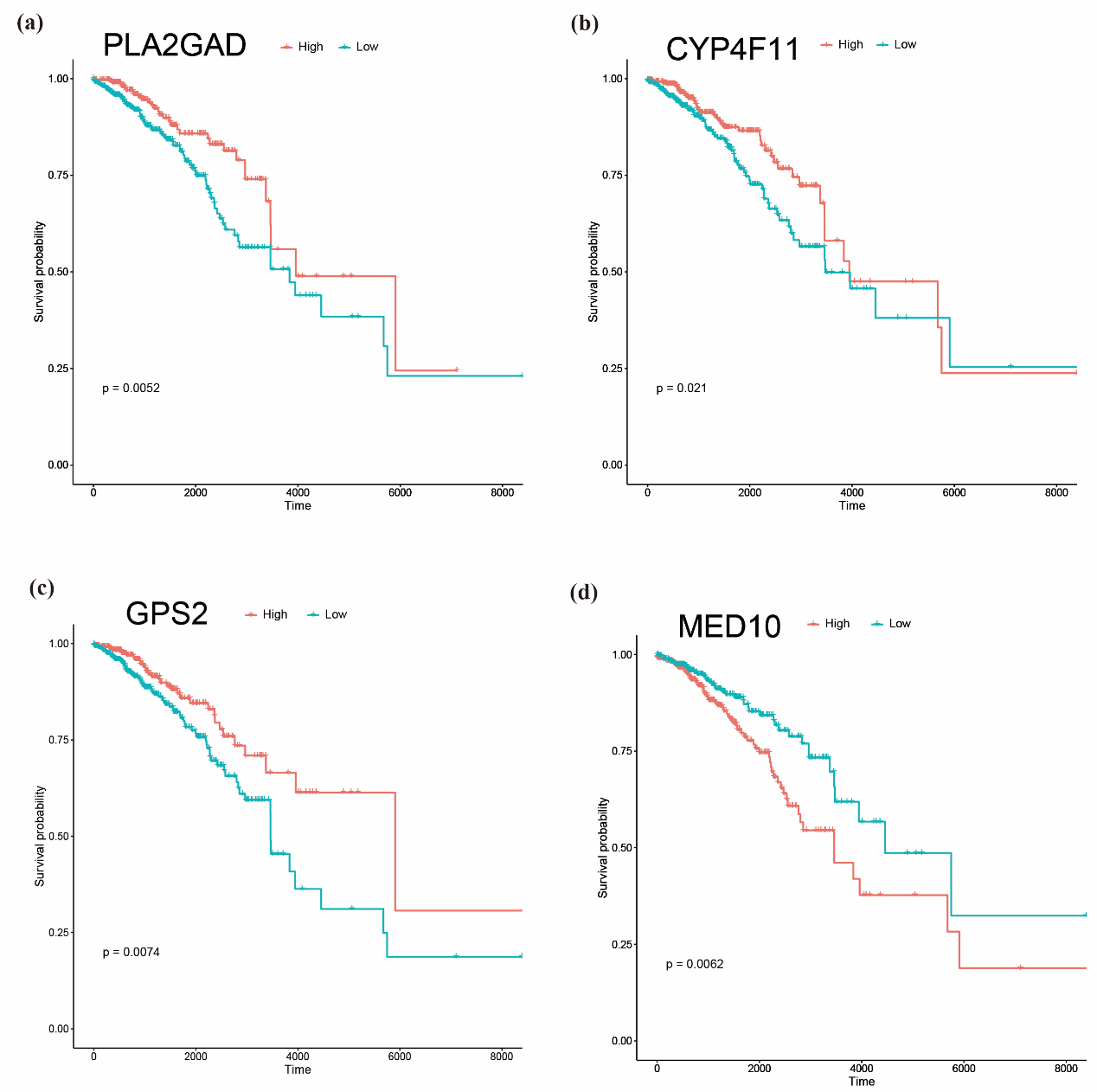


**Supplemental figure 2**. Survival analysis of the four lipid metabolism associated genes for constructing risk model


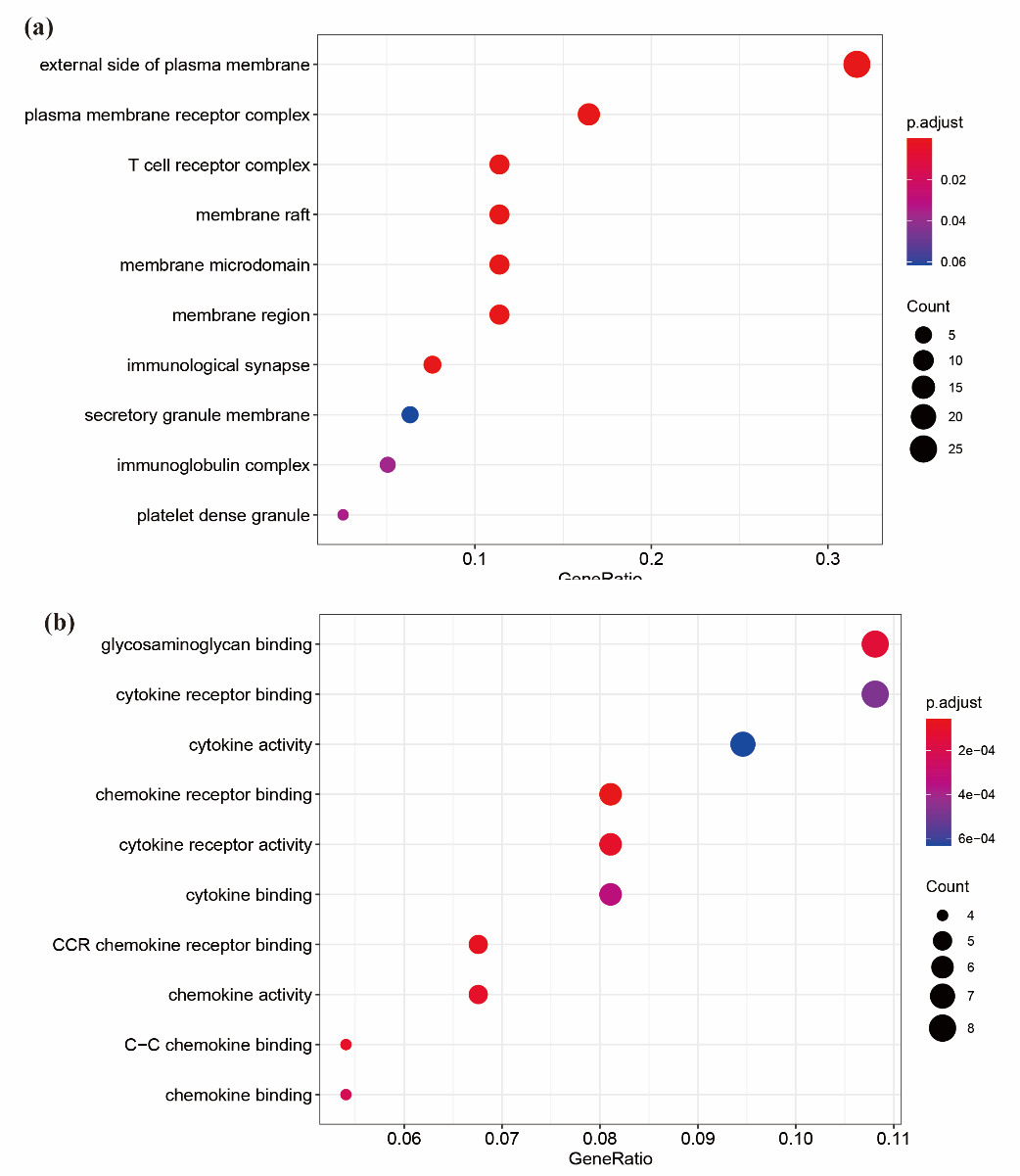


**Supplemental figure 3**. Bubble plots showing the molecular function and cellular component enrichment analysis of the DEGs between the high-risk group and low-risk group.
